# Bone Marrow Fat and Hematopoiesis

**DOI:** 10.3389/fendo.2018.00694

**Published:** 2018-11-28

**Authors:** Huifang Wang, Yamei Leng, Yuping Gong

**Affiliations:** Department of Hematology, West China Hospital, Sichuan University, Chengdu, China

**Keywords:** bone marrow fat, hematopoiesis, leukemia, aplastic anemia, multiple myeloma

## Abstract

Bone marrow fat cells comprise the largest population of cells in the bone marrow cavity, a characteristic that has attracted the attention of scholars from different disciplines. The perception that bone marrow adipocytes are “inert space fillers” has been broken, and currently, bone marrow fat is unanimously considered to be the third largest fat depot, after subcutaneous fat and visceral fat. Bone marrow fat (BMF) acts as a metabolically active organ and plays an active role in energy storage, endocrine function, bone metabolism, and the bone metastasis of tumors. Bone marrow adipocytes (BMAs), as a component of the bone marrow microenvironment, influence hematopoiesis through direct contact with cells and the secretion of adipocyte-derived factors. They also influence the progression of hematologic diseases such as leukemia, multiple myeloma, and aplastic anemia, and may be a novel target when exploring treatments for related diseases in the future. Based on currently available data, this review describes the role of BMF in hematopoiesis as well as in the development of hematologic diseases.

## Introduction

Bone marrow fat (BMF) is located in the bone marrow cavity and accounts for 70% of adult bone marrow volume. It also accounts for approximately 10% of total fat in healthy adults above the age of 25 years (1, 2). Although bone marrow adipocytes (BMAs) are derived from bone marrow mesenchymal stem cells (BMSCs), the origin of BMAs might be heterogeneous (3). BMF has been considered as “inert space filler” for a long period of time (4), and hence its role in the normal development of organisms and in disease has been ignored. More recent work has revealed that BMF plays an important role in energy storage, endocrine function, bone metabolism, and regulation of the growth and metastasis of tumors (2, 5–7). Currently, BMF accumulation is thought to be correlated with osteoporosis, aging, type 1 diabetes, Cushing's disease, estrogen deficiency, anorexia nervosa, and bone metastasis in prostate and breast cancers (8–14). BMF thus is gradually being accepted to play an important role in metabolism.

Bone cavities are predominantly filled with active hematopoietic red bone marrow, the volume of which gradually decreases with age and is subsequently replaced with fat (yellow bone marrow) which gradually fills the entire marrow cavity through dynamic and reversible processes (10, 15, 16). The fat in the bone marrow is different from the subcutaneous and visceral fat and exists in two distinct populations: constitutive marrow adipose tissue (cMAT) and regulated marrow adipose tissue (rMAT). It is hypothesized that cMAT is programmed to develop in a very specific temporal and spatial pattern prior to age 25 and remains preserved upon stress challenges, while rMAT is gradually formed throughout life (17). cMAT contains larger adipocytes, is relatively devoid of active hematopoiesis, and is located in distal skeletal sites (18). In contrast, rMAT is composed of interspersed single adipocytes, and is more closely situated in areas of high bone turnover and is better positioned to actively influence hematopoiesis and/or skeletal remodeling (18). BMF accumulates from birth and happens more rapidly at distal skeletal sites than at proximal skeletal sites. Additionally, the accumulation of BMF over time is consistent with the age-related decline of hematopoietic function (19). Therefore, the decrease of hematopoietic activity in bone marrow with age may be related to the accumulation of BMF.

Some molecules are known to play major roles in the development of BMF. Connective tissue growth factor (CTGF) is a key negative regulator of adipocytic differentiation of BMSCs (20). Activation of the nuclear receptor peroxisome proliferator-activated receptor gamma (PPAR-γ) and fibroblast growth factor 21 (FGF21) promotes adipocyte differentiation but is also known to inhibit osteoblast differentiation (21, 22). On the other hand, activation of the Wnt/β-catenin and Semaphorin 3A (Sema3A) signaling pathways can stimulate BMSCs to differentiate into osteoblasts and inhibit adipogenesis (23, 24). More studies on transcriptional regulators and pathways regulating adipogenesis and osteogenesis are reviewed by Nuttall et al. (25).

BMF constitutes the largest population of cells in the bone marrow cavity, and its relationship with hematopoiesis has attracted further attention in recent years. However, the specific link between BMF and hematopoiesis is not yet clear. In this review, we summarize the relationship between BMF and the hematopoietic microenvironment, hematopoietic stem/progenitor cells, and various lineages involved in the differentiation of blood cells, as well as the role of BMF in the development of hematologic diseases such as leukemia, aplastic anemia, and multiple myeloma.

## Bone marrow fat, hematopoietic microenvironment, and hematopoietic stem/progenitor cells

**Figure 1:**
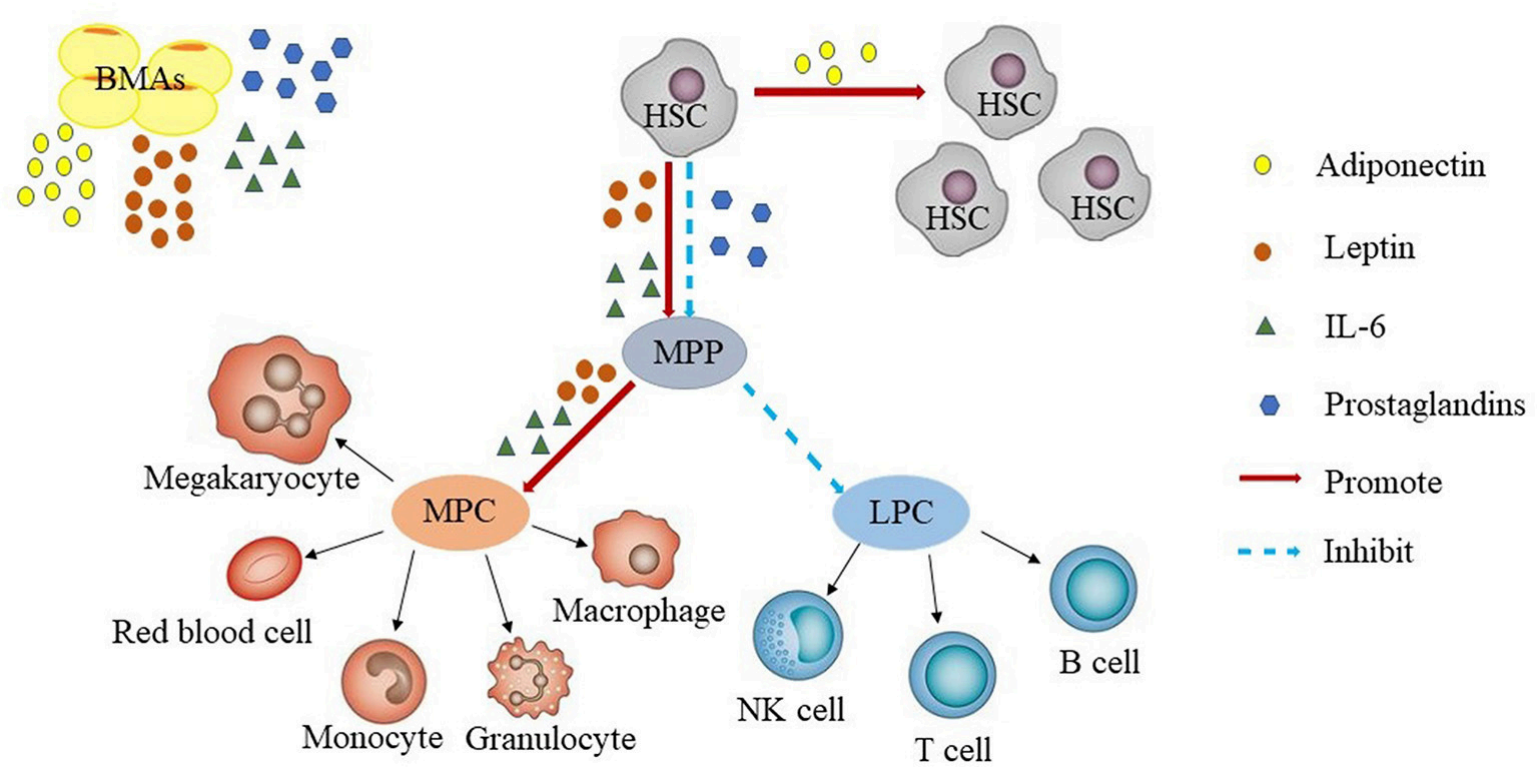
Bone marrow adipocytes and hematopoiesis. BMAs secrete adiponectin, leptin, prostaglandins, IL-6. Adiponectin promotes the proliferation of HSCs. Leptin and IL-6 promotes the differentiation of HSCs, whereas prostaglandins inhibit the proliferation of HSCs. In general, BMAs are more likely to promote HSCs differentiate into myeloid progenitors than into B-lineage progenitors. LPC, lymphoid progenitor cells; MPC, myeloid progenitor cells; HSC, hematopoietic stem cell; LT, long term; MPP, multipotent progenitor; NK, natural killer.

The bone marrow hematopoietic microenvironment, which is also known as the bone marrow hematopoietic niche, consists of marrow stroma cells, the cytokines they secrete, microvessels, and nerves. Intravital microscopy has facilitated intensive study of the bone marrow hematopoietic niche. Using this technique it was found that the bone marrow hematopoietic niche had two distinct states: the homeostatic niche and the reconstituting niche, but the precise definition of these niches remain to be determined (26). The hematopoietic stem cell (HSC) niche is also divided into the endosteal niche and sinusoidal niche. Endosteal niche is localized at the inner surface of the bone cavity, wherein the HSCs are in contact with osteoblasts and might serve as a reservoir for long-term HSCs storage in the quiescent state. The sinusoidal niche, on the other hand, consists of sinusoidal endothelial cell lining blood vessels, which provide an environment for short-term HSCs proliferation and differentiation. Both niches act together to maintain hematopoietic homeostasis (27, 28). Hematopoietic stem/progenitor cells further differentiate into two major categories of myeloid progenitor cells and lymphoid progenitor cells. Myeloid progenitor cells have the potential to differentiate into the myeloid lineage, while lymphoid progenitor cells have the potential to differentiate into lymphoid sub-lines (Figure 1).

The bone marrow stroma is a mixture of mesenchymal stem/progenitor cells and their progenies, including adipocytes, osteolineage cells, pericytes, chondrocytes, and fibroblasts (29). BMAs, which are the most abundant type of cells in the bone marrow hematopoietic microenvironment, play a vital role in maintaining balance between proliferation and differentiation of hematopoietic stem/progenitor cells. Spindle-shaped N-cadherin^+^CD45^−^ osteoblastic cells are arranged on the surface of the bone and constitute a major component of the niche supporting HSCs (30). HSCs are maintained and regulated by various signals and cell types of the surrounding microenvironment. These cell types include the vascular sinusoidal endothelial cells, perivascular BMSCs, mature hematopoietic cells, and non-myelinating Schwann cells. Among these cells, the vascular sinusoidal endothelial cells and perivascular BMSCs support the self-renewal of HSCs by secreting the cytokines chemokine stromal cell-derived factor CXCL12 and stem cell factor (SCF) that play important roles in hematopoiesis, spermatogenesis, and melanogenesis (31). On the other hand, mature hematopoietic cells and non-myelinating Schwann cells are involved in HSC quiescence and localization through various pathways, including the TGF-β and CXCL4 signaling pathways (29). Additionally, osteoblasts, BMSCs, and mature hematopoietic cells support multipotent and committed progenitors and play a crucial role in efficient lymphopoiesis, myelopoiesis, and erythropoiesis (29). BMF gradually accumulates with age; this is accompanied by a decrease in HSCs (32). However, it remains unclear whether there is a direct connection between these two phenomena, and this issue needs further exploration.

The technological advancement of three-dimensional electron microscopy allows the observation of BMAs and their relationship with surrounding tissues. Three-dimensional electron microscopy has revealed that BMAs display hallmarks of metabolically active cells, including polarized lipid deposits, dense mitochondrial networks, and areas of endoplasmic reticulum. Triacylglycerol droplets containing fatty acids are taken up and/or released at three key areas: at the endothelial interface, in the hematopoietic milieu, and on the bone surface (33). This directly demonstrates at the microstructure level that bone marrow adipose tissue is active and is not “inert space filler.” In the hematopoietic environment of the proximal tibia, BMAs interact extensively with maturing cells of the myeloid lineage and are closely associated with erythroblast islands (33), providing a spatial basis for the interaction between BMF and HSCs.

BMF, as a part of the hematopoietic niche, affects HSCs proliferation and differentiation by secreting adiponectin, leptin, prostaglandins, IL-6, and other adipocyte-related derived factors (34–41). However, so far, it is not clear, whether these factors are also derived from sources other than BMF and contribute to the effects on HSCs. Adiponectin is a protein hormone which is involved in regulating glucose levels as well as fatty acid breakdown. In humans, it is encoded by the ADIPOQ gene and is produced in the adipose tissue (42). Adiponectin promotes the proliferation of HSCs and maintains their undifferentiated state. HSCs increased through adiponectin were more efficient at hematopoietic reconstitution in lethally irradiated mice through AdipoR1-mediated signaling (34). Leptin a 16-kDa protein produced by adipocytes, is also known to be secreted by BMF in the bone marrow microenvironment, resulting in high concentrations of this protein in the bone marrow (43, 44). Additionally, multiple isoforms of the leptin receptor (LEPR) have been identified, including the long isoform and several isoforms with short cytoplasmic domains (45, 46). Leptin independently or synergistically promotes the proliferation of HSCs (35–38), prostaglandins inhibit HSCs through induction of apoptosis (39, 40), and IL-6 promotes the differentiation of HSCs (41) (Figure 1).

The specific role of BMF in regulating the differentiation of HSCs and other bone marrow lineages has not been clarified to date. A study by Tavassoli et al. found that hematopoiesis in the bone marrow of rabbits was enhanced after removal of BMF (47). Naveiras et al. showed that in the adipocyte-rich bone marrow of the mouse tail vertebrae, number of adipocytes from the thoracic to the caudal vertebrae increased while hematopoiesis gradually decreased. Furthermore, this was accompanied with reduced percentage of multipotent, common myeloid, granulocyte-monocyte, and megakaryocyte-erythroid progenitors (48). This data indicated a predominantly negative influence of adipocytes on hematopoiesis within the bone marrow microenvironment. Furthermore, the absence of adipocytes in fatless A-ZIP/F1 mice rescued hematopoiesis in the tail, while the use of the PPARγ inhibitor bisphenol-A-diglycidyl-ether (BADGE) prevented bone marrow adipose formation in bone marrow-transplanted mice, enhanced hematopoiesis, and shortened recovery time after hematopoietic transplantation (48). These data indicated that BMF inhibits bone marrow hematopoiesis. However, it is interesting to note that the HSCs in the caudal vertebrae were quiescent, not senescent, and that they were able to grow faster on exposure to suitable environment (48). Further they also observed that HSCs purified from the caudal vertebrae had higher long-term engraftment rates (48, 49). Despite these data, contrary views on the effect of BMF on HSCs have emerged ceaselessly. BMAs have been reported to reappear on the 7th day after radiation injury, which corresponds to the initiation of hematopoietic proliferation, therefore, BMAs potentially support HSCs (50). Moreover, bone marrow adipocytes have been found to inhibit HSCs differentiation and prolong their survival *in vitro* (51). BMAs supported hematopoiesis in the homeostatic state *in vitro* but had no effect on the same *in vivo* (52). A study by Zhou et al. revealed that BMF from irradiated and non-irradiated mice produced high amounts of SCF, an essential cytokine that plays a pivotal role in HSCs maintenance, which promoted hematopoietic recovery after 5-fluorouracil treatment or irradiation (53). Strikingly, genetic deletion of SCF from adipocytes inhibited hematopoietic regeneration after myeloablation, whereas genetic deletion of the same cytokine from other important cells present in the niche (osteoblasts and endothelial or hematopoietic cells) did not affect hematopoietic recovery after 5-fluorouracil treatment or irradiation. Therefore, SCF secreted by BMF was necessary for the maintenance of hematopoiesis (53). Additionally, it was found that the role of BMF differed in various compartments of the bone marrow in mice. Adipocytes in the tail vertebrae inhibited hematopoiesis by inhibiting angiogenesis in the bone marrow niche after radiation, whereas adipocytes in long bones promoted hematopoietic recovery after radiation, despite two locations acting as an important source of SCF (53). As an important component of the HSC niche, BMF modulates HSC function, but the role of BMF in hematopoiesis remains controversial. For example, Naveiras et al. found that BMF significantly inhibits hematopoiesis, and Zhou et al. found that BMF promotes hematopoiesis by secreting SCF. The role of BMF on the hematopoietic microenvironment and on hematopoietic stem/progenitor cells may be distinct *in vivo* and *in vitro*, in different species, or in different locations in the same individual, indicating that the connections between BMF and bone marrow hematopoiesis are extremely intricate. In the future, there will be more like-minded scholars cooperating to explore the links between BMF and hematopoiesis.

## Bone marrow fat, myelopoiesis and lymphopoiesis

BMF gradually accumulates with age and a high-fat diet (10, 54), and is accompanied by a decrease in B-lineage progenitor cells, whereas HSCs are more likely to differentiate into myeloid progenitors than into B-lineage progenitors (54–56), suggesting that BMF may be associated with myelopoiesis and lymphopoiesis. BMF secretes adipocyte-derived soluble factors *in vitro* that inhibit B lymphopoiesis, particularly at the stage at which lymphogenic progenitor cells differentiate into pre-proB cells, and simultaneously promotes the differentiation and subsequent proliferation of HSCs into the myeloid lineage (57). Similarly, it is shown that BMF had a negative effect on the early stages of B lymphocyte proliferation in the bone marrow of elderly people (57). Kennedy et al. revealed that BMF induces the production of myeloid-derived suppressor cells (MDSCs), particularly in mononuclear cells (CD11b^+^ Ly6C^+^ Ly6G^−^), which inhibit B lymphopoiesis by producing IL-1 (58). At the same time, BMAs activate the inflammasome, such as the nod-like receptor 3 (NLRP3), and directly inhibit B lymphopoiesis (59). Inflammasome activation is also likely to promote thymic degeneration (60, 61) and exert a negative effect on T-lymphocyte proliferation (62). Blocking the NLRP3 inflammasome with glybenclamide inhibited the accumulation of MDSCs and boosted B lymphopoiesis *in vitro* (59). Furthermore, the deletion of NLRP3 in mice prevented thymic atrophy and the decline of T lymphopoiesis (62). BMF induces the production of multipotent progenitors by the bone marrow and promotes HSCs differentiation toward the myeloid lineage. It also induces the secretion of granulocyte-colony stimulating factor, monocyte-colony stimulating factor, and granulocyte monocyte-colony stimulating factor by bone marrow stromal cells, thereby negatively regulating B-lineage cell production and lymphopoiesis, and promoting myelopoiesis (58). In rabbits, the inflammatory cytokine S100A9 increases with the accumulation of BMF in the bone marrow and induces the expression of IL-6, TNF, and IL-1β in bone marrow myeloid cells. However, it is not clear whether the increase of S100A9 is directly related to the accumulation of BMF (59). Naveiras et al. showed that removal of adipocytes from bone marrow in mice led to an increase in hematopoiesis, including B lymphopoiesis (48). Studies on the toxicant tributyltin (TBT) showed that TBT exposure induces bone marrow adipogenesis and activates PPARγ in the bone marrow, resulting in a decrease in peripheral B lymphocytes (63). Thiazolidinediones such as troglitazone and the tyrosine analog GW7845 have been shown to activate PPARγ and induce pre-B apoptosis by upregulating NF-κB (64). Adiponectin secreted by BMF in young rabbits could negatively and selectively influence lymphopoiesis by inducing prostaglandin synthesis (40). This effect was most apparent in early lymphoid progenitors, and cyclooxygenase inhibitors were shown to abrogate the response of early lymphoid progenitors to adiponectin in stromal cell-containing cultures (40). Another BMF adipokine, leptin, has the opposite effect on lymphopoiesis (37). Leptin promotes differentiation and proliferation of the lymphoid lineage, and is also helpful in promoting myelopoiesis (36). In *db/db* mice, wherein the LEPR is truncated, the steady-state levels of peripheral blood B cells and CD4-expressing T cells are dramatically reduced (36, 38). However, there are other theories regarding the role of BMF in myelopoiesis. BMF hampered granulopoiesis through neuropilin-1(NP-1)-induced granulocyte-colony stimulating factor inhibition and dexamethasone-induced multinuclear granulocyte proliferation by the downregulation of NP-1 (65). Preadipocytes in the bone marrow therefore appear to contribute to granulopoiesis during the fibrocytic stage and become inactive during hematopoiesis when they are converted to adipocytes (66). The study by Naveiras et al. also found that BMF suppresses bone marrow hematopoiesis, including myelopoiesis (48). An exploration of the mechanism underlying the side effect of rosiglitazone on the bone marrow demonstrated that it inhibits myeloid differentiation of HSCs after stress. Rosiglitazone exerts this effect partially by inducing bone marrow adipogenesis and by targeting the bone marrow microenvironment. The selective PPARγ antagonist BADGE may be able to partially reverse these effects, however, PPARγ is also expressed in myeloid cells with profound effects, and some of these effects could also be cell-autonomous in myeloid cells (49). Thus, so far, the role of BMF in myelopoiesis is complex, still unclear, and controversial and may be mediated by multiple mechanisms of action.

## Bone marrow fat and erythropoiesis

As early as 1978, Ambika et al. revealed through a study on a group of rabbits that the long-term use of phenylhydrazine induces hemolysis and stimulates erythropoiesis, which in turn stimulates the lipolysis of BMAs (67). This suggested, for the first time, that BMF is associated with erythropoiesis and may be involved in metabolic processes that support hematopoietic function. BMF accumulation leads to anemia in patients with reduced leg loading, which may impair hematopoiesis in two ways: first, by occupying hematopoietic space, and second, by directly interfering with hematopoiesis via paracrine action within the bone marrow microenvironment (68). Robles et al. used a three-dimensional electron microscope to observe the bone marrow of rodents and found that BMAs interact with the phagocytic reticular macrophages of the erythroblast islands, which provided a spatial basis for the role of BMAs in erythropoiesis (33). It has been estimated that a single BMA is capable of interacting with more than 100 hematopoietic cells through both direct cell-cell contact and indirect signals via binding with the core macrophage of erythroblast islands (33, 69, 70). Erythrocytes develop and mature in the erythroblast islands (69). Immature islands are often distant from bone marrow sinusoids and migrate toward the sinusoid when the erythrocytes mature (69). Therefore, BMAs may help deliver energy to distant, immature red blood cell islands, thereby supporting the maturation of red blood cells. This is consistent with previous animal experiments in which the sizes of BMAs were shown to rapidly reduce during active erythropoiesis after phenylhydrazine-induced anemia or severe blood loss (67, 71). A recent article reported that the use of erythropoietin to stimulate high-fat diet-fed mice caused an increase in the hematocrit values accompanied by a decrease in bone marrow adipose tissue and the disappearance of adipose tissue (72). However, more research is still needed in the future to identify the signaling pathway/s linking BMF and erythropoiesis.

## Bone marrow fat and hematologic diseases

### Bone marrow fat and leukemia

In normal hematopoiesis, the LEPR is expressed in CD34^+^ cells, and leptin was found to induce the proliferation and differentiation of these cells (73). Primary acute promyelocytic leukemia (APL) cells express high levels of the long isoform of the LEPR. BMAs produce membrane-bound leptin that participates in the bone marrow cytokine network, regulate the proliferation, survival, and apoptosis of APL cells via direct cell-to-cell contact, and prevent APL cells from drug-induced apoptosis (74). Connective tissue growth factor promotes the differentiation of BMSCs into adipocytes, which produce leptin in the bone marrow, thereby promote leukemic cell engraftment and growth within the bone marrow niche (20).

BMF protects acute lymphoblastic leukemia (ALL) cells from apoptosis induced by various chemotherapeutic agents, although the mechanism of protection is not yet known (75, 76). Subsequent studies demonstrated that ALL cells induce an oxidative stress response in adipocytes, which promotes the resistance of ALL cells to daunorubicin, an anthracycline antileukemia drug (77, 78). Adipocytes confer dexamethasone (a cortical hormone drug which is often used to treat chronic lymphocytic leukemia) resistance to chronic lymphocytic leukemia cells by providing lipid factors. BMF supports the survival and proliferation of acute myeloid leukemia (AML) blast cells (79). A possible mechanism for this may be the induction of lipolysis of triglycerides stored within BMAs into fatty acids, which are then released into the bone marrow microenvironment in a process dependent on the chaperone protein fatty acid binding protein-4 (80). Ultimately, fatty acids are metabolically beneficial for the survival and proliferation of AML cells (80). Recent studies have investigated the correlation between BMA morphology and the prognosis of patients with AML. These studies have confirmed that in AML patients, an increase in small BMAs, rather than total BMAs, is associated with poor prognosis (81). Lu et al. demonstrated that growth differentiation factor 15 secreted by leukemia cells induced the transformation of large BMAs into small BMAs, which in turn could promote leukemic cell growth (82). Almost at the same time, other researchers reported opposite findings—that a decrease in adipocyte volume in patients with complete remission from AML is closely related to long-term recurrence-free survival. Growth differentiation factor 15, which is secreted by marrow mononuclear cells in response to chemotherapy and partially blocks adipogenesis, may exert synergistic effects on strengthening chemotherapeutic efficacy and may be used in predicting good outcomes for patients with AML during complete remission (83). These observations suggest that AML interrupts adipogenesis in red bone marrow, leading to impaired myelo-erythroid maturation (84). *In vivo*, administration of PPARγ agonists have been shown to induce bone marrow adipogenesis, rescuing healthy hematopoietic maturation and repressing leukemic growth (84). These seemingly contradictory conclusions suggest that more rational experiments are needed to explore the role of GDF1 in adipogenesis and AML. Searching for a signaling pathway that disrupts the interaction between leukemic cells and adipocytes may be considered a new approach for targeted therapy against leukemia and combating drug resistance.

### Bone marrow fat and multiple myeloma

BMF plays a role in the proliferation, apoptosis, and migration of multiple myeloma (MM) cells in the bone marrow microenvironment (85). However, BMAs disappear during disease progression, while other stromal cells (endothelial cells, fibroblasts) are still present and are activated. This suggests that the role of BMAs is mainly limited to the initial stage of the disease before the remodeling of the bone marrow microenvironment occurs (85). BMAs are the only cells that secrete leptin in the MM microenvironment, and the addition of leptin leads to a slight increase in the proliferation of MM cells *in vitro*, which participate in these processes by affecting diffusion (85). Leptin serum levels are elevated in patients with MM at the time of diagnosis, but these levels did not increase with the progression of MM. Moreover, leptin levels decreased after treatment (86). Studies have found that the expression of LEPRs on MM cells can predict the response of patients to thalidomide treatment (87). BMF upregulates the expression of autophagic proteins in MM cells by secreting adipocyte-derived factors, such as leptin and resistin, that leads to the suppression of caspase cleavage and apoptosis, and ultimately protect MM cells from chemotherapy-induced apoptosis (88). Resistin protects MM cells from chemotherapy-induced apoptosis by inhibiting chemotherapy-induced caspase cleavage via the NF-κB and phosphoinositide 3 kinase (PI3K)/Akt signaling pathways and by enhancing the expression of ATP-binding cassette (ABC) transporters in myeloma cells via demethylation of the ABC gene promoter (89). However, resistin is secreted not only by BMF but also by monocytes, macrophages, spleen, and bone marrow cells (90). Therefore, further studies are needed to differentiate the effect of resistin secreted by the BMF from the effect of resistin secreted by other stromal cells on myeloma growth and survival (89).

### Bone marrow fat and aplastic anemia

Aplastic anemia (AA) is a complex bone marrow failure syndrome characterized by extremely hypoplastic bone marrow and peripheral blood pancytopenia. One of the key pathogenic factors for AA is the alteration of the hematopoietic microenvironment (91). It is known that the osteogenesis and adipogenesis of BMSCs are well balanced in normal bone marrow, and that disrupting this balance leads to disease (92, 93). Interestingly, in the bone marrow of patients with AA, the number of adipocytes has been observed to be higher, while the number of osteoblasts is lower (94). Thus, the reduction of these cells would affect normal hematopoiesis. Clinical studies have suggested that arsenic trioxide (ATO) is clinically effective in treating patients with AA (95, 96). Furthermore, studies have demonstrated that BMSCs from patients with AA are prone to differentiation into adipocytes rather than into osteoblasts *in vitro* (97, 98), and that treatment with arsenic trioxide could partially restore the unbalanced differentiation of BMSCs (98). This suggests that arsenic trioxide administration, which improves the balance between osteogenic and adipogenic differentiation, may be a novel therapeutic approach for AA. miRNA-204 has been shown to inhibit osteogenic differentiation and promote adipogenesis of BMSCs by directly inhibiting Runx2, providing a novel therapeutic method for the treatment of diseases with unbalanced osteogenic and adipogenic differentiation (98). Wnt signaling inhibits the differentiation of BMSCs into adipocytes (99). A Wnt signal activator combined with cyclosporine A has been shown to be more effective in treating AA than cyclosporine A only in mouse models, implying that Wnt signaling could inhibit the differentiation of bone marrow BMSCs into adipocytes and improve bone marrow hematopoiesis (100). This confirms the importance of BMF in the pathogenesis of AA. The transcription factor GATA-2 is expressed in HSCs and early hematopoietic progenitors and plays a crucial role in hematopoiesis (101). A decrease in GATA-2 level affects the proliferation and survival of HSCs (102, 103). GATA-2 mRNA level was found to be significantly lower in AA patients than in normal individuals (104, 105). GATA-2 is also expressed in preadipocytes and inhibits terminal differentiation into mature adipocytes by suppressing PPARγ (106). GATA-2 expression was significantly reduced in BMSCs in patients with AA, whereas PPARγ expression was significantly higher in AA patients than in normal subjects (107). Therefore, GATA-2 participates not only in the generation and maintenance of HSCs but also in the regulation of the hematopoietic microenvironment (108). Identifying the mechanisms by which GATA-2 regulates HSCs and bone marrow BMSCs may be useful in developing novel therapeutic approaches for bone marrow failure syndrome.

## Conclusion

BMAs in the hematopoietic microenvironment influence the hematopoietic process through space support, production of derived cells (MDSCs), and secretion of adipocyte-related derived factors (adiponectin, leptin, prostaglandins, and IL-6). The exact role of BMF in hematopoiesis is not yet clear because of the fractious connection between BMF and other cells in the hematopoietic microenvironment as well as hematopoietic stem/progenitor cells. The heterogeneity of HSCs indicates that the hematopoietic niche supporting HSCs function may have a matching heterogeneity potential (109). As an important part of the HSC niche, it is not clear whether BMF is heterogeneic and how it plays a role in different hematopoietic niches such as the endosteal niche and the sinusoidal niche. It is important to explore the role of different BMAs in hematopoiesis, including the location and species of BMF in future studies. It is also meaningful to explore the effect of BMF in hematopoiesis in different hematopoietic states, such as the homeostatic state and stressing state, *in vitro and vivo*. By exploring the link between BMF and the neighboring cell populations in the hematopoietic niche, the current understanding of the complex relationship between BMF and hematopoiesis may be improved. Research on the interactions between adipocyte-derived factors and other signaling factors in the bone marrow microenvironment and their role in hematopoiesis and hematologic diseases will facilitate the discovery of new methods in the field of hematological diseases.

## Author contributions

HW and YL contributed to writing, and YG contributed to editing this work.

### Conflict of interest statement

The authors declare that the research was conducted in the absence of any commercial or financial relationships that could be construed as a potential conflict of interest.
